# Rapid Knockout and Reporter Mouse Line Generation and Breeding Colony Establishment Using EUCOMM Conditional-Ready Embryonic Stem Cells: A Case Study

**DOI:** 10.3389/fendo.2015.00105

**Published:** 2015-06-30

**Authors:** James L. J. Coleman, Karen Brennan, Tony Ngo, Poornima Balaji, Robert M. Graham, Nicola J. Smith

**Affiliations:** ^1^Molecular Cardiology Program, Victor Chang Cardiac Research Institute, Darlinghurst, NSW, Australia; ^2^St. Vincent’s Clinical School, University of New South Wales, Darlinghurst, NSW, Australia; ^3^BioCORE, Victor Chang Cardiac Research Institute, Darlinghurst, NSW, Australia

**Keywords:** EUCOMM, conditional knockout, Cre recombinase, Flp recombinase, mouse model, inducible knockout, C57BL/6

## Abstract

As little as a decade ago, generation of a single knockout mouse line was an expensive and time-consuming undertaking available to relatively few researchers. The International Knockout Mouse Consortium, established in 2007, has revolutionized the use of such models by creating an open-access repository of embryonic stem (ES) cells that, through sequential breeding with first *FLP1* recombinase and then Cre recombinase transgenic mice, facilitates germline global or conditional deletion of almost every gene in the mouse genome. In this Case Study, we describe our experience using the repository to create mouse lines for a variety of experimental purposes. Specifically, we discuss the process of obtaining germline transmission of two European Conditional Mouse Mutagenesis Program (EUCOMM) “knockout-first” gene targeted constructs and the advantages and pitfalls of using this system. We then outline our breeding strategy and the outcomes of our efforts to generate global and conditional knockouts and reporter mice for the genes of interest. Line maintenance, removal of recombinase transgenes, and cryopreservation are also considered. Our approach led to the generation of heterozygous knockout mice within 6 months of commencing breeding to the founder mice. By describing our experiences with the EUCOMM ES cells and subsequent breeding steps, we hope to assist other researchers with the application of this valuable approach to generating versatile knockout mouse lines.

## Introduction

The humble laboratory mouse has had a home in scientific research since the 15th century, and continues to prove an indispensable resource for investigators wishing to unravel the biology of mammalian development, genetics, physiology, and pathology. This is largely due to the mouse’s small size and resource demand, short generation time, and suitability for breeding within a laboratory environment (1). With the development of “gene targeting” technologies using mouse embryonic stem (ES) cells in the late 20th century, researchers gained the ability to selectively mutate the mouse genome at loci of their choice (2). However, the promises of this technology are dependent on a high level of expertise in targeting vector design, ES cell isolation, and culture and surgical embryo implantation. Moreover, this process is time consuming and expensive. Clearly, researchers who are eager to commence animal studies would benefit from a library of pre-mutated mouse ES cells; a need that was addressed last decade with the establishment of the International Knockout Mouse Consortium (IKMC).

The IKMC was established in 2007 with the aim of mutating all protein-coding regions of the mouse genome using gene targeting and gene trapping in C57BL/6 mouse ES cells (3). The IKMC is itself a consortium of partner programs: Knockout Mouse Project (KOMP) (USA), European Conditional Mouse Mutagenesis Program (EUCOMM) (Europe), EUCOMM: Tools for Functional Annotation of the Mouse Genome (EUCOMMTOOLS) (Europe), North American Conditional Mouse Mutagenesis Project (NorCOMM) (Canada), and Texas A&M Institute for Genomic Medicine (TIGM) (USA) (4). Prioritization of targets has been based upon community demand, expert committees, and the perceived importance of individual genes, with little overlap between programs (3). In our case, both of our genes of interest were targeted and available through EUCOMM, so we will focus this review on the gene targeting approach specific to EUCOMM ES cells. For more information on the development of the IKMC and gene targeting, we refer the reader to Capecchi (2), Skarnes et al. (5), and Bradley et al. (4). As of 2012, more than 17,400 ES cell lines had been generated by the IKMC for targeted gene deletion (4).

## EUCOMM Gene Targeting Strategy

**Figure 1:**
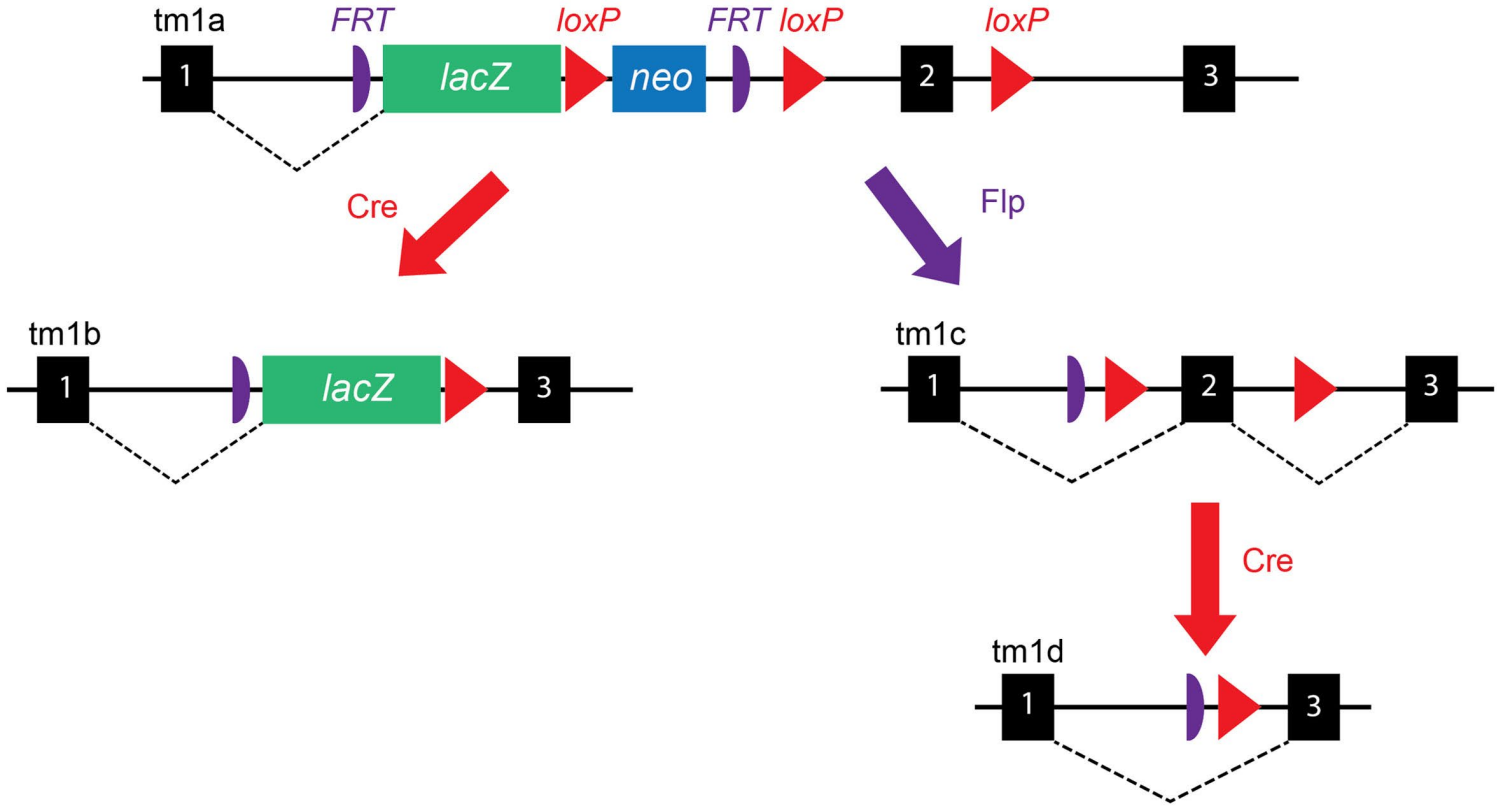
**EUCOMM targeting vector construction**. Schematic of the EUCOMM vector design illustrating the targeting and recombination process for a prototypical gene with three exons (black boxes). See text for description. Figure adapted from Skarnes et al. (5).

One of the challenges faced by the IKMC was the design of simple and broadly applicable targeting vectors. The main EUCOMM strategy has been to develop “knockout-first” conditional allele targeting, a powerful approach that allows both reporter-tagging and conditional mutation of a given gene-of-interest (GOI). Development of the various targeting vectors is described in detail in the original paper (5); here, we provide an outline of how this approach would be applied to a prototypical GOI containing three exons with exon 2 having been identified as “critical” for gene function. Shown in Figure 1, an *IRES:lacZ* trapping cassette (internal ribosome entry site upstream of *lac Z*) is placed 5′ of a *loxP*-flanked, promoter-driven, neomycin-resistance selection cassette, which lies immediately upstream of exon 2. The neomycin-resistance cassette facilitates *in vitro* selection of targeted ES cells, while the *lacZ* cassette can be used in the generation of reporter-tagged animals, i.e., animals expressing lacZ in the tissues that express the GOI. A third *loxP* site is inserted immediately after exon 2 to facilitate its removal and therefore generation of experimental knockout animals in which both alleles of the GOI have been inactivated; hence, they are homozygous *null* for the GOI. To allow simultaneous removal of both the *lacZ* and neomycin-resistance cassettes, two *FRT* (flippase recognition target) sites are inserted in the allele; one upstream of the *lacZ* cassette and one between the neomycin-resistance cassette, and the *loxP* site immediately before exon 2. This allele is referred to as “targeted mutation 1a” (*tm1a*), and is designated “knockout-first,” because the insertion of the *lacZ* trapping allele is itself expected to disrupt splicing of the GOI, although both the neomycin-resistance and *lacZ* cassettes should be removed before assessment of the knockout phenotype (see below).

A key feature of the EUCOMM system is that the vector design is versatile and high-throughput. As such, EUCOMM employed automated gene annotation and a computer-assisted vector design pipeline (5), whereby the 5′-most “critical” exon is identified and targeted for deletion – a critical exon is one that will lead to a frameshift if deleted, is common to all transcript variants and will disrupt >50% of the protein-coding region of the GOI (4). Once identified, an algorithm was used to choose 50-mers suitable for recombineering and placement of *loxP* sites in intronic regions where they are unlikely to disrupt transcription. While this approach has enabled the EUCOMM team to target thousands of GOIs, this targeting system can only be applied to approximately 40% of protein-coding genes (5). Genes with just one or two exons, those lacking an exon that is common to all isoforms, or where alternative 5′ transcripts exist, must be targeted using alternative methods (generated by the other members of the IKMC). Although mice bearing the *tm1a* allele will effectively have their GOI deleted, additional breeding steps are needed to create appropriate reporter or knockout experimental animals, as outlined below and in Figure 1.

### Generation of lacZ reporter mice

In addition to targeted gene silencing, the elegant EUCOMM allele design features the potential to replace GOI translation with expression of a β-galactosidase enzyme (from the *lacZ* cassette), providing a means to ask *where* the GOI is normally expressed. β-galactosidase expression can be assessed at the resolution of tissues and cell types, using either histochemical staining or immunohistochemistry (6). The advantage of performing immunohistochemistry to detect the β-galactosidase protein, instead of the native GOI product, is that researchers can employ well-established and sensitive β-galactosidase immunohistochemistry protocols, instead of having to design a protocol for their own GOI, which in many cases may not have been subjected to detailed evaluation and may be heavily dependent on the availability of high quality antibodies. This is particularly important for G protein-coupled receptors, for example, where the specificity of “specific” antibodies is often poor, as shown by similar staining patterns in both wild type and knockout mice (7–9).

By crossing *tm1a* mice with a global “Cre deleter” mouse [a transgenic B6.C-Tg(CMV-Cre)1Cgn/J (The Jackson Laboratory, USA, Stock number 006054) mouse expressing Cre recombinase ubiquitously under the control of a human cytomegalovirus (*CMV*) promoter], recombination at *loxP* sites results in deletion of both the neomycin cassette and exon 2, leading to the generation of *tm1b*, *lacZ* reporter mice that express β-galactosidase in cells where the GOI would ordinarily be expressed (Figure 1). The *Cre* is X-linked in this mouse line due to random integration of the transgene into the X chromosome, but expression of Cre recombinase precedes X-inactivation, thus gene excision will proceed efficiently in both male and female mice (10). Where offspring inherit both a *tm1a* allele and a *Cre* allele, *loxP* sites are cut and recombined, removing the neomycin-resistance selection cassette and exon 2 of the GOI, while preserving the *lacZ* cassette.

### Generation of “floxed” mice

As a first step toward generating the knockout line and to also restore GOI expression to that of a pseudo-wild type animal, heterozygous *tm1a* mice are crossed with transgenic C57BL/6J mice expressing an enhanced variant of *Saccharomyces Cerevisiae FLP1* recombinase (FlpE) in all tissues, under the human β-actin promoter (transgenic B6.Cg-Tg(ACT*FlpE*)9205Dym/J, available from The Jackson Laboratory, USA, Stock number 005703) (11). Where offspring inherit both the *tm1a* allele and the *FlpE* allele, the *FRT*-flanked region of *tm1a* is excised and recombined, removing both the *lacZ* and neomycin-resistance cassettes and restoring GOI expression. These mice are referred to as *tm1c* or “floxed” mice and serve as both the founder line for the generation of knockout mice and as the control line for experiments. *tm1c* mice are essentially “wild type” as the only difference between their allele and that of wild type C57BL/6 mice is the introduction of two *loxP* sites flanking exon 2; however, this should be confirmed experimentally in the unlikely event that the *loxP* sites have disrupted gene splicing or an important regulatory or enhancer element within the introns.

### Generation of knockout mice

Finally, breeding of *tm1c* with either the same Cre deleter mouse as for *tm1b*, or alternatively with a tissue-specific or inducible Cre deleter mouse (discussed in the next section), leads to the generation of “*tm1d”* mice, i.e., animals in which exon 2 of the GOI has been deleted and the residual gene transcript is likely degraded as a result of nonsense-mediated decay (Figure 1). A more detailed description of the breeding strategies we employed to generate each line is outlined later.

## Advantages and Disadvantages of the EUCOMM System

### EUCOMM mice do not require backcrossing because they use C57BL/6 ES cells

Historically, early gene targeting efforts were performed using ES cells derived from various 129 substrains, largely because they are easier to work with and have resulted in high rates of germline transmission. However, few studies are now performed using the 129 background because these mice breed poorly, are susceptible to testicular teratomas, and are known to display other phenotypic abnormalities, e.g., behavioral changes (12). Instead, germline transmission is often tested on the preferred C57BL/6 mouse background, as these mice are better breeders, are the reference strain for the first mouse genome (13), are the background for many Cre modifying transgenic mice, and are widely used throughout the research community because of their marked and well-characterized responses to many disease models^1^ (e.g., diet-induced obesity, diabetes, atherosclerosis). Moreover, if germline transmission is established using 129 ES cells, the resultant mice must be further crossed to C57BL/6 mice for 10 generations for the line to be considered congenic [note that the genes located very close to the GOI will likely still be from the 129 strain and may lead to “passenger gene” effects (14)]. A significant advance in the field, brought about in large part due to the efforts of the IKMC and EUCOMM, is the generation of thousands of knockout-first alleles using C57BL/6 ES cells (15). By using these cells to generate chimeras, researchers can obviate the need for time- and resource-demanding backcrossing and be assured that their mice have a pure genetic background.

### J vs N: What’s in a name?

The use of C57BL/6 ES cells is undoubtedly one of the major selling points of the EUCOMM system, along with the ability to produce conditional-ready knockout-first mice. However, one should be aware of subtle differences between sub-strains of C57BL/6 mice. In developing the JM8 ES cells used for the EUCOMM pipeline, Pettitt et al. preferred the less common C57BL/6N sub-strain to the C57BL/6J because the 6N cells demonstrated superior growth and morphology when compared to their 6J cousins (15). Yet, the genetic background of the FlpE and Cre deleter mice required for subsequent EUCOMM breeding steps is usually C57BL/6J [e.g., B6.Cg-Tg(ACT*FlpE*)9205Dym/J, stock number 005703; B6.C-Tg(CMV-Cre)1Cgn/J, stock number 006054; and B6.FVB(129)-Tg(Myh6-Cre/Esr1*)1Jmk/J, stock number 005657; available from The Jackson Laboratory, USA], meaning that subsequent generations of these mice would effectively represent backcrossing to a C57BL/6J genetic background. Thus, even after 10 generations of crossing to C57BL/6J mice, a small confounding section of C57BL/6N genetic material would remain around the selected allele, estimated at ~20 cM (16). C57BL/6J and C57BL/6N have been separated for at least 220 generations (as of 2013) and until recently were characterized by relatively few genetic differences, the most common being a five exon deletion in the nicotinamide nucleotide transhydrogenase (Nnt) gene in C57BL/6J mice, resulting in altered insulin secretion and glucose tolerance (17, 18). Minor phenotypic differences have also been noted, such as retinal degeneration in C57BL/6N mice (19) and behavioral differences between the C57BL/6 sub-strains (20). More recently, however, next generation sequencing of both sub-strains revealed 34 coding single nucleotide polymorphisms (SNPs), 2 coding small indels (insertions/deletions), 146 non-coding SNPs, and 54 non-coding small indels (21). Furthermore, using the broad and carefully standardized EMPRESSslim phenotyping pipeline, Simon et al. identified 27 phenotypic features that were significantly different between the two sub-strains and that were consistently found to differ even when performed at multiple research centers (21). These differences were in categories such as ophthalmology, cardiovascular health, metabolism, behavior, clinical chemistry, hematology, and immune function (21). Given the increased recognition of such phenotypic differences, careful design of animal breeding strategies and interpretation of experimental results are required.

### Choice of Cre recombinase mouse gives temporal and spatial flexibility

One of the most powerful aspects of the EUCOMM system is the capacity to modify gene deletion in a tissue- or time-specific manner by breeding *tm1c* mice with specialized Cre recombinase lines. While a global *tm1d* mouse can be generated by crossing *tm1c* with the *CMV*-driven Cre recombinase mice outlined above, there are many cases in which global deletion is not ideal. This is best illustrated in a recent study by the Sanger Institute Mouse Genetics Project that described the careful phenotyping of 489 targeted alleles from the EUCOMM system, where 42% of genes were found to be essential for viability (29% lethal, 13% sub-viable) (22). In such cases, or where a developmental defect confounds investigation of an adult condition, an inducible Cre recombinase would allow deletion of the GOI after the mouse has reached adulthood. For the inducible transgenes, Cre recombinase is most commonly fused to a modified estrogen receptor that preferentially binds tamoxifen instead of estrogen (23). The Cre recombinase is constitutively expressed but remains in the cytoplasm, physically separated from the floxed gene of interest. Upon binding to tamoxifen, Cre recombinase now translocates to the nucleus where it excises floxed genes by recombination at *loxP* recognition sites (23).

Another powerful example of the utility of the EUCOMM approach is the ability to decipher the tissue-specific physiology that underpins a phenotype seen in a global gene deletion, either in an inducible or constitutive manner, by using cell type- or tissue-specific promoters to drive Cre expression. For example, angiotensin II (AngII) has long been known to contribute to blood pressure homeostasis via the ubiquitously expressed angiotensin type 1 (AT_1_) receptor. Elegant studies by Tom Coffman and colleagues, in which they cross-transplanted kidneys from wild type and AT_1_ knockout mice, demonstrated that renal AT_1_ receptors are critical for maintaining baseline blood pressure (24) and are necessary for AngII-mediated cardiac hypertrophy (25). However, the AT_1_ receptor is expressed in multiple cell types within the kidney, so further studies required cell-type specific Cre deletion of the AT_1_ receptor to further understand the mechanism of these findings. Subsequent studies with either PEPCK-Cre (phosphoenolpyruvate carboxykinase-Cre) (26) or inducible KAP2-Cre (kidney androgen-regulated protein-Cre) (27) driven excision of the AT_1_ receptor from the renal proximal, but not distal, tubules demonstrated that it was this sub-population of cells responsible for the observed phenotype. The incredible flexibility afforded by the EUCOMM conditional-ready mice is underlined by the availability of more than 2000 Cre mouse strains (not all are live stock); suitable mice can be searched for using the Cre portal at the Mouse Genome Informatics website^2^ or the International Mouse Strain Resource^3^.

### EUCOMM mice still require multiple generations of breeding to remove contaminating transgenes and the neomycin selection cassette

**Figure 2:**
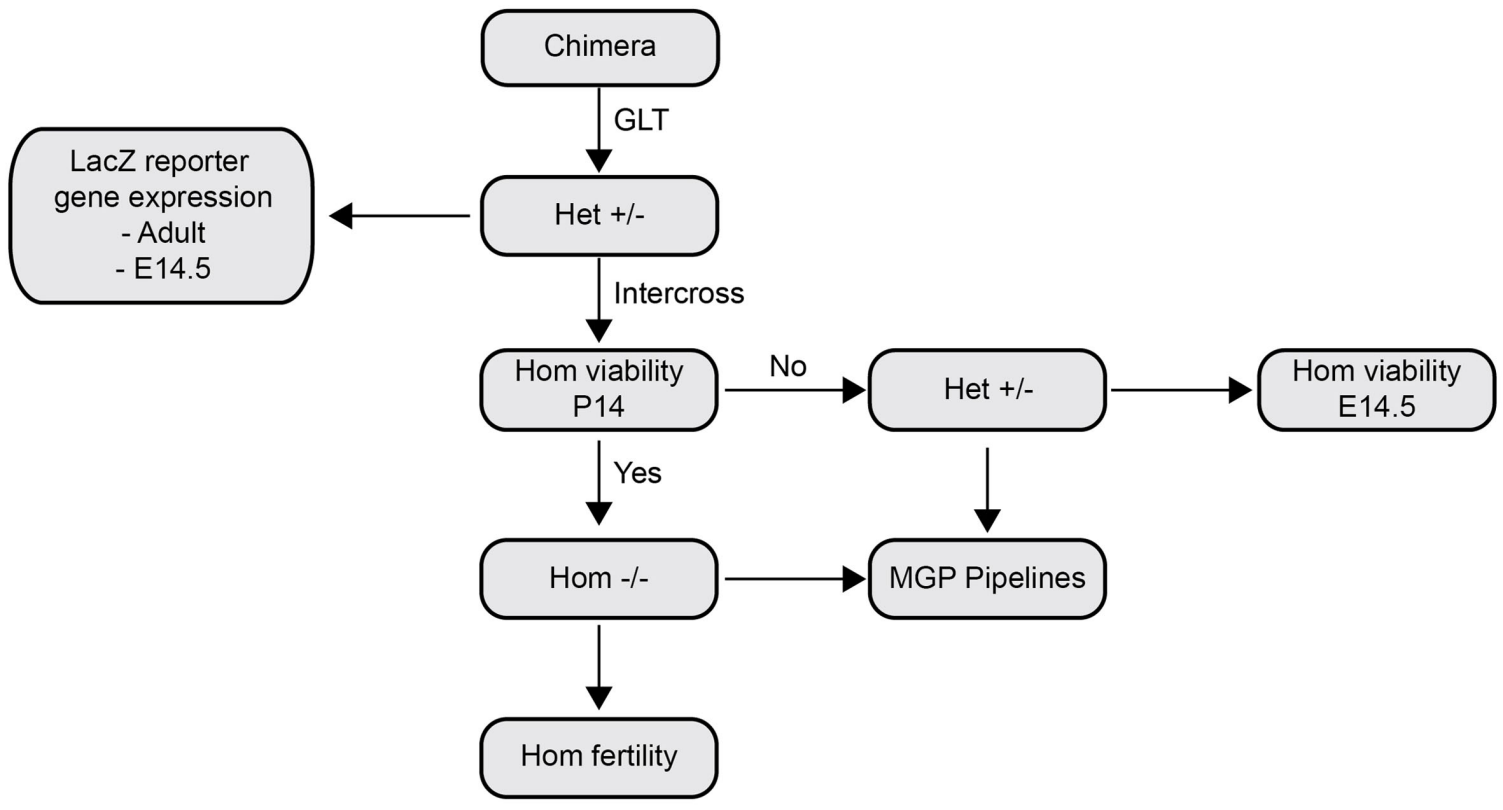
**Outline of phenotyping protocol for EUCOMM mice used by the Sanger Institute Mouse Genetics Project**. The Sanger Institute Mouse Genetics Project describes a standardized series of tests applied to novel EUCOMM lines. Chimeric mice are outcrossed with C57BL/6 to determine germline transmission (GLT) and heterozygous *tm1a* mice intercrossed to assess viability at 14 days after birth. If viable, homozygous *tm1a* mice enter the Mouse Genetics Project (MGP) standardized phenotyping pipeline or are tested for fertility. In the case of embryonic lethality or poor survival, heterozygous mice are used for the MGP pipeline and homozygous embryos examined at E14.5 to determine the cause of sub-viability. In parallel to the intercross protocol, a subset of heterozygous *tm1a* mice are crossed to Cre recombinase mice to generate *lacZ* reporters. Figure adapted from White et al. (22).

As discussed above, the use of C57BL/6 ES cells obviates the need for 10 generations of backcrossing to achieve genetic homogeneity if the target genetic background is also C57BL/6. Furthermore, the fact that the *tm1a* mice are considered to be knockout-first means that initial phenotyping can be performed immediately and without further breeding steps. This is particularly advantageous for larger scale phenotyping exercises such as the Sanger Institute Mouse Genetics Project (22), which used brother–sister matings of *tm1a* heterozygotes to generate homozygous “nulls” for their phenotyping screen (see Figure 2 for pipeline). In general, however, it is desirable to remove both the neomycin cassette (by crossing with FlpE mice to generate *tm1c*) and any remaining recombinases (either FlpE or Cre) before phenotyping the knockout mice, since both have been linked to phenotypic effects independent of those due to inactivation of the GOI. For example, Cre recombinase can introduce DNA alterations via cryptic *loxP* sites in the mammalian genome (28), with pathogenic consequences reported in the gastrointestinal (29), respiratory (30), and cardiovascular systems (31, 32), to name a few. Similarly, retention of antibiotic selection cassettes, such as those used in the EUCOMM system, can cause direct disruption of gene regulatory elements and unexpected disruption of genes far downstream from the initial mutation. Strikingly, Pham et al. (33) report disruption of genes more than 100 kb downstream of the phosphoglycerate kinase (PGK)-Neo cassette site within the granzyme B cluster (33). Similarly, insertion of PGK-Neo in the human β-globin locus control region was sufficient to induce at least a twofold reduction in the expression of all genes in the locus, but, importantly, this phenomenon was found to be reversible upon subsequent removal of the cassette (34). Thus, despite EUCOMM mice being “knockout-first,” we believe it is essential to breed the mice for several generations to achieve knockout and floxed mice that will be free of interference from exogenous sequences.

## Case Study: Generation of Two Different Knockout Mouse Lines Using EUCOMM ES Cells

Like most research groups, we wanted to be able to generate our transgenic mouse lines in the quickest and most efficient manner possible. In this section, we describe the generation of each of the lines outlined above for two GOIs and the breeding strategies employed. All animal studies were performed according to the *Australian Code for the Care and Use of Animals for Scientific Purposes*, 8th Edition (2013) and were approved by the Garvan Institute of Medical Research/St Vincent’s Hospital Animal Ethics Committee under project numbers 11/26 and 13/30.

### Germline transmission from EUCOMM ES cells

We outsourced the initial generation of *tm1a* heterozygotes to the Monash University Embryonic Stem Cell-to-Mouse (ES2M) facility, an Australian Government-subsidized fee-for-service initiative designed to provide live IKMC mice to academic researchers.^4^ Due to the facility’s size, expertise, and collaborations, the generation of transgenic mice via ES2M is both time- and cost-efficient, and subject to stringent quality control. The descriptive statistics describing critical parameters in this process, from ordering ES cell clones to the generation of *tm1a* heterozygotes for our two GOIs, is displayed in Table 1. For a repository-wide description of germline transmission rates for KOMP mice, we refer the reader to www.komp.org/gltrates.php.

**Table 1:** **Germline transmission of genes of interest using EUCOMM ES cells**.

Clone	Karyotype	Injected	# injections	# chimeras	Chimeras mated					
					Injection #	%	# litters	# pups	# hets	# litters for hets
GOI-1#1	15/20 40, Y	Yes	3	21 (5F)	1	M 90	5	29	0	–
					1	M 75	4	63	0	–
					1	M 60	0	0	–	–
					1	M 55	7	63	0	–
					1	M 50	8	74	0	–
					3	M 95	0	0	–	–
					3	M 80	8	68	5	1
					3	M 65	8	56	0	–
GOI-1#2	Abnormal karyotype	No	–	–	–	–	–	–	–	–
GOI-1#3	Abnormal karyotype	No	–	–	–	–	–	–	–	–
GOI-1#4	16/20 40, Y	No – not targeted	–	–	–	–	–	–	–	–
GOI-2#1	10/20 40, Y	No, low karyotype	–	–	–	–	–	–	–	–
GOI-2#2	Clone unavailable	–	–	–	–	–	–	–	–	–
GOI-2#3	15/20 40, Y	Yes	2	0	–	–	–	–	–	–
GOI-2#4	13/20 40, Y	Yes	4^a^	4	2	M 65	0	–	–	–
					3	M 70	5	49	21	1
					3	M 60	1	12	0	–
					4	M 60	2	17	0	–

Hets, heterozygotes.

*^a^Note that only three injections were required for germline transmission. The fourth injection was performed in parallel as a back-up*.

Based upon past experience and anecdotal evidence that germline transmission may be more difficult to achieve with C57BL/6 ES cells, ES2M staff recommended four ES cell clones per GOI be purchased from EUCOMM (35). For GOI-1, we found that only one of the four clones passed quality control (two clones had an abnormal karyotype and one clone did not actually contain the *tm1a* allele). Of the only clone with a suitable karyotype for microinjection, three rounds of injections were required to achieve germline transmission – of 21 chimeras, eight showed sufficient coat-color chimerism to warrant breeding with C57BL/6J mice to test for germline transmission and only one of these chimeras produced heterozygote offspring. Thus, 5/354 pups carried our transgene of interest (1.4% success).

For GOI-2, only three of four clones were available for shipping and one clone had ≤50% euploidy and therefore failed the necessary criteria for injection (35). GOI-2 clone #3 was first chosen for microinjection because of its favorable karyotype but failed to produce live births from either injection round. In contrast, GOI-2 clone #4 had a lower karyotype but resulted in four chimeras, albeit from four rounds of injections. Fortuitously, one of the four chimeras showed germline transmission, producing 21 offspring (26.9% success).

On average, the time from ES2M order submission to arrival of heterozygous *tm1a* mice at our animal facility was 28 months. It is important for researchers to accommodate at least 4 months from the time of order for receipt of the ES cells from EUCOMM, a common processing time for the international consortia (35). In our case, we experienced a longer time to germline transmission than most because of the quality of our ES cells – while KOMP-derived ES cells are routinely subjected to quality control screening before dispatch, this has only recently been implemented by EUCOMM (35). As outlined in Table 1, only three of the eight ordered clones were of passable quality, the remaining being either morphologically or karyotypically abnormal or incorrectly targeted. In contrast to the >2 years to germline transmission, breeding and expansion of the lines was rapid.

### Receipt of *tm1a* founders and expansion of the breeding colony

**Figure 3:**
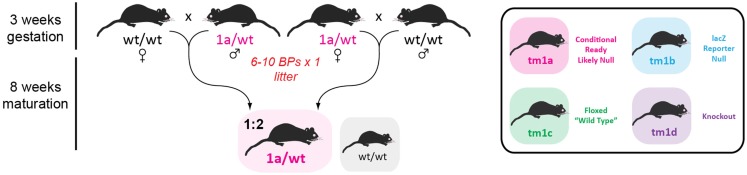
**Initial colony expansion of founder mice**. Both male and female *tm1a* heterozygotes were outcrossed with C57BL/6J partners to increase our breeding colony for subsequent line generation. Listed on the left is the time taken from establishing a new breeding pair (BP; numbers indicated in red) to the time when offspring are sexually mature (7–8 weeks of age). Mice genotypes enclosed in gray boxes are those surplus to requirements, while the colored box indicates the desired genotype and the expected Mendelian ratio. Inset: key used for remaining figures.

We received 5 GOI-1 (3 male, 2 female) and 12 GOI-2 (5 male, 7 female) *tm1a* mice from the Monash University ES2M facility and decided to immediately expand our colony in a single breeding step, shown in Figure 3, to ensure we had enough *tm1a* heterozygotes for subsequent line generation. As described in Table 2, we set up 10 breeding pairs (BPs) for GOI-1 and six for GOI-2, so that within one generation (3 weeks gestation and 8 weeks to reach sexual maturity) we had established colonies with the expected Mendelian inheritance ratios (indicating that our GOIs did not affect heterozygous animal viability). Any subsequent litters were used for cryopreservation and reserves (see below). Also discussed below, careful monitoring of all litters produced is critical given that the *tm1a* mice are knockout-first and the mice may have unexpected phenotypes that adversely affect their welfare.

**Table 2:** **Breeding statistics for the first generation of each EUCOMM line**.

Line	Strategy (female × male)	# BPs	# litters per BP	Total # pups reported	% Weaned males	Av. litter size	Pre-wean mortality (%)	Genotype % (expected %)		Genotype by sex (% desired genotype)	
								Desired	Other	Male	Female
**GOI-1**											
*tm1a*	*tm1a*/wt × wt or wt × *tm1a*/wt	10	2	139	61.2	5.8	5.0	54.1 *tm1a* (50)	45.9 wt (50)	58.8	46.5
*tm1b*	*tm1a*/wt x Cre/Y	8	1–2	88	0^a^ (52.3)	7.3	1.1	36.1 *tm1b* (25)	63.9 *tm1a* or Cre only (75)	n.a.	36.6
*tm1c*	Flp/wt x *tm1a*/wt	10	1–2	120	57.9	6.6	16.8	26.5 *tm1c* (25)	73.5 *tm1a* or Flp only (75)	29.5	23.1
*tm1d*	Cre/Cre x *tm1c*/wt	7	1–2	80	38.7	7.3	0	34.2 *tm1d* (50)	65.8 wt (50)	32.2	35.4
**GOI-2**											
*tm1a*	*tm1a*/wt x wt or wt x *tm1a*/wt	6	1–4	64	100^a^ (43.7)	6.4	0	72.2 *tm1a* (50)	27.8 wt (50)	72.2	n.a.
*tm1c*	Flp/Flp x *tm1a*/wt or *tm1a*/wt x Flp/Flp	8	1	54	46.3	7.7	0	53.7 *tm1c* (50)	46.3 wt (50)	62.5	50
inducible *tm1d*	*tm1c*/wt; Flp/wt x MCM/MCM	4	1–3	53	37.7	6.6	1.9	25.7 *tm1d and* MCM (25)	74.3 wt or Flp (75)	28.6	23.8

BP, breeding pair.

*^a^Indicates that only one sex was weaned. % of total males is indicated in parentheses*.

### Generation of lacZ reporter mice for characterization of gene expression patterns

**Figure 4:**
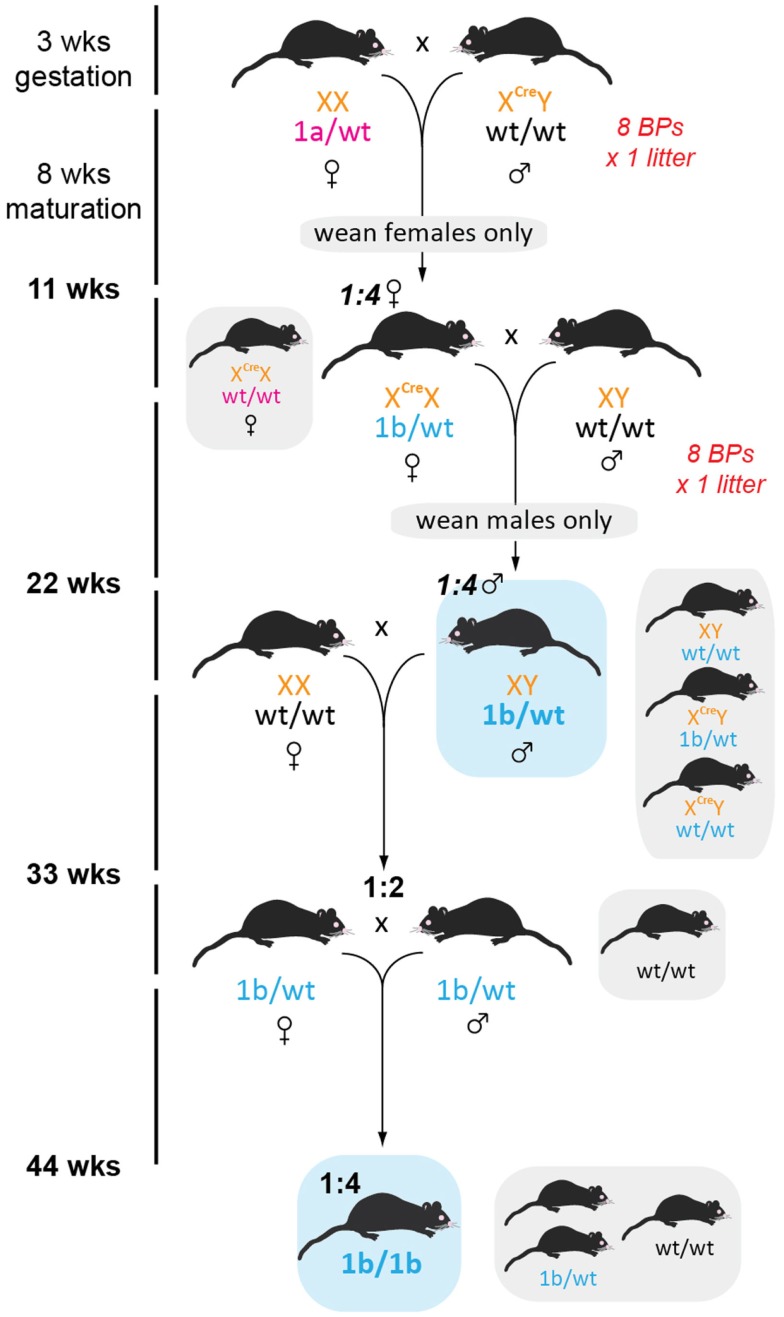
**Generation and maintenance of *lacZ* reporter mice**. Male *CMV*-Cre mice were crossed with female *tm1a* heterozygotes and all female offspring genotyped for conversion to *tm1b* (all females must be heterozygous for *Cre*). The recombinase was then removed by crossing *tm1b* convertants to C57BL/6J males and all females culled prior to weaning because they potentially carry *Cre*. Recombinase negative males were then used for experiments or used in further breeding before heterozygous × heterozygous breeding pairs could be established. Homozygous *lacZ* mice can then be used for timed matings and embryo harvest. Note that *tm1b* mice are themselves knockout mice, so should be monitored accordingly for adverse phenotypic effects. Mice in gray boxes are surplus to requirements.

Generation of *tm1b*, or *lacZ*, reporter mice required a two-step breeding strategy to achieve recombinase-free *lacZ* heterozygotes within 6 months. Outlined in Figure 4 and Table 2, eight BPs were established involving male *Cre* hemizygotes on a congenic C57BL/6 background (10) crossed to *tm1a* females to produce a single litter and male pups were culled prior to weaning, thus ensuring that all remaining offspring were heterozygous for *Cre*. After genotyping for *tm1a* to *tm1b* conversion, positive females were outcrossed to wild type C57BL/6J males and then all female offspring culled – we adopted this conservative approach of culling all females when only half would be *Cre* positive because it was the only way to ensure *Cre* was removed from our line (in contrast to genotyping, where a negative result could reflect a genotyping failure, rather than the absence of *Cre*; given the known effects of Cre contamination, this was not a risk we were prepared to take). At this stage, *Cre* negative *tm1b* mice can be used for characterization of GOI expression, or as breeders for colony establishment. Traditionally, a single copy of the *lacZ* transgene is sufficient for β-galactosidase staining or immunohistochemistry. It should be noted, however, that these mice are effectively heterozygous knockouts and should be monitored accordingly.

### Floxed mice generation and simultaneous removal of FlpE

**Figure 5:**
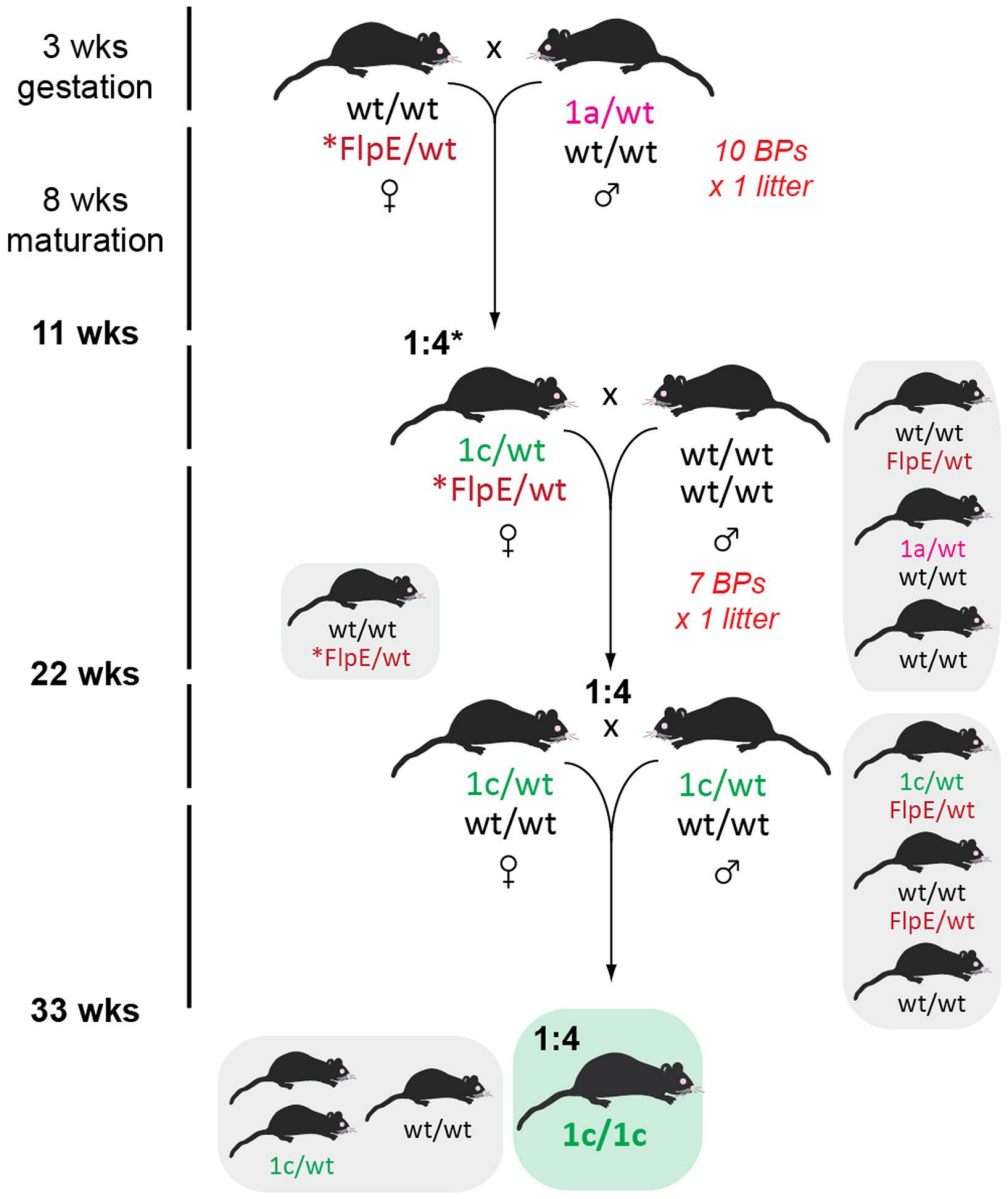
**Floxed mice generation**. Female FlpE recombinase heterozygotes (or homozygotes in the case of GOI-2, indicated by an asterix) were crossed with male *tm1a* heterozygotes and resultant *tm1c*-converted mice identified by genotyping (*1:2 conversion for GOI-2). The floxed mice were subsequently outcrossed and *tm1c* heterozygotes free of contaminating FlpE recombinase were chosen for brother × sister matings and subsequent line maintenance.

Floxed, or *tm1c*, mice were generated by crossing with FlpE recombinase transgenic mice on a congenic C57BL/6J background (11). For GOI-1, we had yet to establish a homozygous colony of FlpE mice, so heterozygous × heterozygous pairs were established, while GOI-2 homozygous × heterozygous breeding was possible later (Figure 5). About eight or 10 BPs were established and in both cases the expected Mendelian ratio of *tm1c* conversion was observed (Table 2). A subset of offspring carrying the *tm1c* allele was outcrossed to wild type C57BL/6J mice and floxed heterozygotes, negative for *FlpE*, were then interbred to establish mice for a subsequent two-tiered stem and expansion colony management strategy, as per Brennan (16).

### Rapid generation of knockout mice

**Figure 6:**
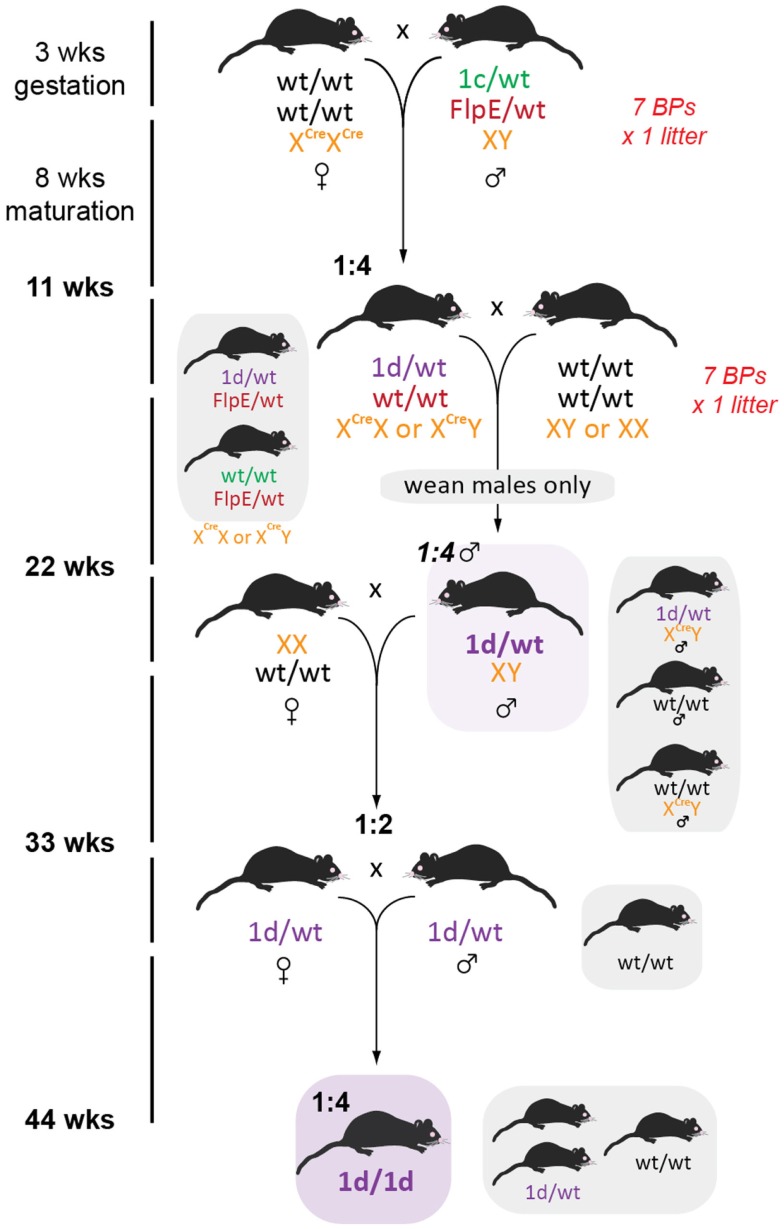
**Generation of global knockout mice**. Female homozygous *CMV*-Cre mice were crossed with *tm1c* males such that all offspring bearing both *tm1c* and *Cre* alleles will convert to *tm1d*. To hasten removal of the FlpE recombinase, only *FlpE* negative offspring were retained and then crossed to wild type C57BL/6J partners. Because *CMV*-Cre is X-linked, all female pups were culled, resulting in a 1:4 ratio of desired recombinase-negative *tm1d* males. Subsequent outcrossing to wild type C57BL/6J females generated 50% *tm1d* heterozygotes, which were then intercrossed to generate the homozygous knockout line.

To establish a colony of knockout mice (*tm1d*) free from both FlpE and Cre recombinases in the shortest time possible, we took seven *FlpE* positive floxed males and set up BPs with seven *Cre/Cre* females. Resultant offspring had a 50% conversion rate to *tm1d* (as per *tm1a* heterozygotes, we did not observe lethality due to gene deletion) and *FlpE* negative male or female littermates were bred for a subsequent generation (Figure 6). Because the *Cre* recombinase is X-linked in this line, all females from this generation were heterozygous for *Cre* and were therefore culled prior to weaning to remove the recombinase from the colony. Male offspring were genotyped for the presence of either the *tm1d* allele or *Cre* recombinase; only *tm1d*^+^/*Cre*^-^ mice were retained. While the present breeding scheme has ensured that we have generated heterozygous knockout mice within 6 months of receiving our founder *tm1a* mice, by using homozygous *Cre* female breeders and then culling all female offspring we have necessitated an additional outcrossing step before we can establish *tm1d* heterozygous × heterozygous BPs and therefore test our homozygous knockouts. Using a heterozygous *Cre* female at this mating step and then screening for *Cre* by genotyping would shorten the time taken to achieve the desired genotype by a further generation, or 11 weeks.

### Generation of inducible knockout mice within a year

**Figure 7:**
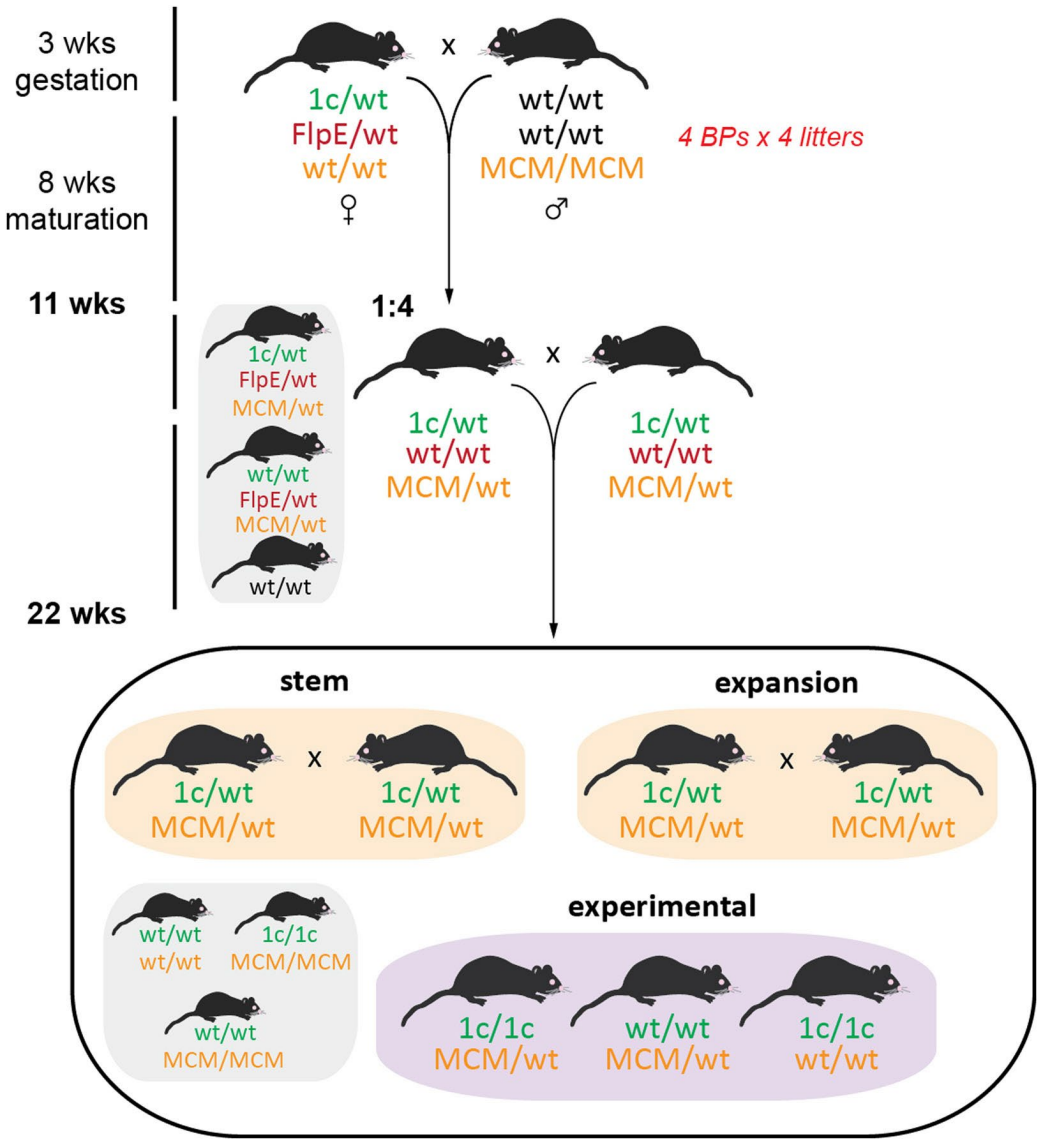
**Initial breeding scheme for generation of MerCreMer inducible knockout mice**. At the same time as generating the *tm1d* knockout mice, we also established an inducible cardiomyocyte-specific deletion line. Female *tm1c* mice were bred to homozygous α-*MHC* *MerCreMer* (MCM) C57BL/6J males; the *tm1c* allele was selected for by PCR and remains unconverted (requires tamoxifen administration for Cre induction), while all *FlpE* positive offspring were culled at this step. Brother × sister matings were established as the stem breeding scheme for future generations, while expansion breeding pairs comprised *tm1c* heterozygotes that were either wild type or homozygous for *MCM* – the expansion pairs will yield all necessary experimental mice as well as feed back to the stem colony. Stem and expansion breeding is discussed in Ref. (16).

One of the primary motivators for using the EUCOMM model was our desire to generate inducible, tissue-specific knockout mice so that we could explore the physiological roles of our GOIs without potentially confounding developmental influences. One such conditional model is the cardiomyocyte-specific deletion of our GOIs by crossing *tm1c* heterozygotes with homozygous B6.FVB(129)-Tg(Myh6-Cre/Esr1*)1Jmk/J (stock number 005657, available from The Jackson Laboratory) (36). This mouse line expresses a tamoxifen-inducible Cre recombinase (MerCreMer*)* directed by the mouse cardiac-specific α-myosin heavy chain (α*-MHC*) promoter. Excision is initiated by administration of tamoxifen, which binds to the modified estrogen receptor (Mer; recognizes tamoxifen but not estrogen) to facilitate translocation of Cre from the cytoplasm to the nucleus, where it is able to remove the *loxP*-flanked exon 2 of the GOI. Only one copy of *Cre* is necessary for efficient transgene excision while mice must be homozygous for *tm1c*, necessitating multiple breeding steps (Figure 7).

## Additional Considerations

### Animal welfare and monitoring

**Figure 8:**
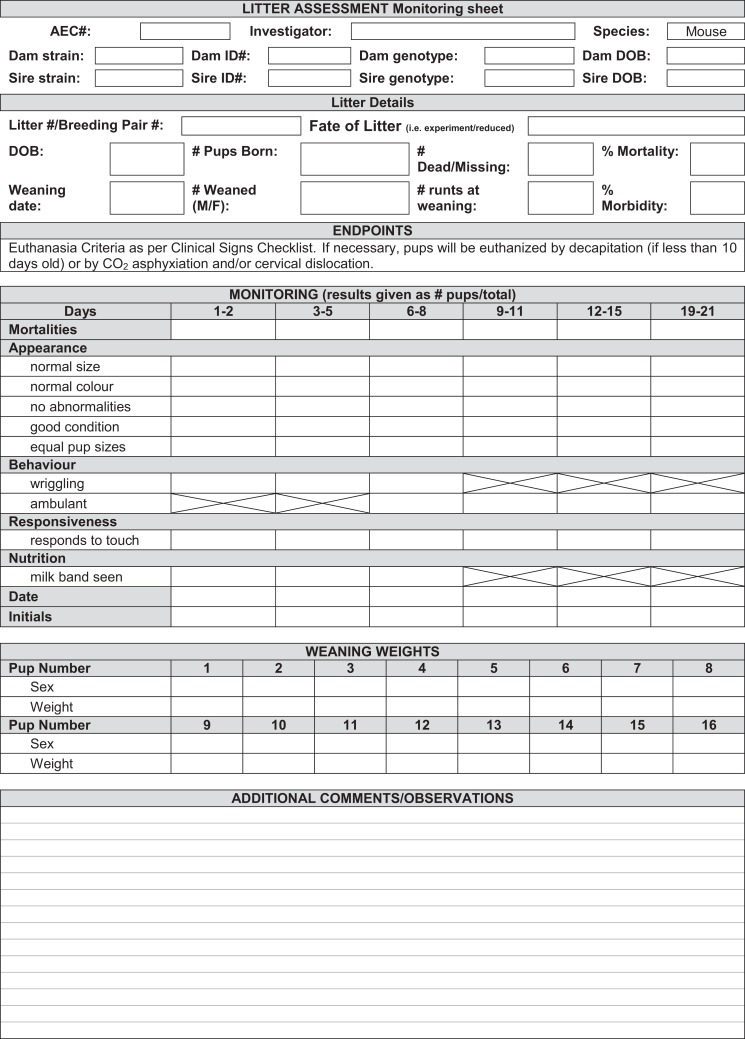
**Sample sheet for animal welfare monitoring of EUCOMM mice**. An example of the kind of litter monitoring sheet that should be used to assess the welfare of new lines, accounting for mortality, abnormalities or differences in appearance, behavior, responsiveness, and nutrition. Boxes with a strike-through are not applicable to that particular age.

In most cases, the phenotype of *tm1a* and *tm1d* mice is either unknown or presumed, necessitating careful monitoring of mice throughout initial breeding steps to screen for any adverse phenotypes that might arise. Aside from experimental interest and monitoring for embryonic lethality, this is paramount for the welfare of the animals as early identification of detrimental effects can alleviate distress. A number of parameters should be assessed upon receipt of adult *tm1a* animals (16) and upon the birth of the first litter of all other mouse lines that will be generated “in-house” from the EUCOMM system. We devised a specific monitoring scheme, adapted from Brennan (16) and shown in Figure 8, and found that all lines developed in the course of this case study were free of adverse effects or overt phenotypic differences. It is important to note that at this stage of the breeding scheme, we do not know the genotype of the offspring until after weaning. For expediency, we chose to monitor litters without this information, instead focusing on gross developmental differences within and between litters. Effects of viability of a particular genotype were later dismissed when we were able to confirm that all lines displayed expected Mendelian inheritance ratios (Table 2).

For litter monitoring, neonates were observed every 2 days from birth until weaning (3 weeks). Mortality was assessed and found to be minimal except for the GOI-1 *tm1c* line (Table 2), where several litters were destroyed (because this line should be as close to wild type as possible, and the fact that no other phenotypic differences were observed, we attribute this to the young age of the mothers). Neonates were normal and equal in size relative to one another, of the expected coat color, free of morphological abnormalities, and had a healthy body condition. As per expected developmental trajectories, pups were ambulant 7–8 days after birth (and exhibited wriggling prior to that time) and responded to touch. Milk bands were visible on neonates until approximately 7–8 days after birth. Weaning and mature mice had body weights and growth rates similar to that of their parental strain. Animal coats were free of hair loss, skin color was as expected, and mice exhibited normal posture and gait. Behaviorally, mice were alert and responsive. No clinical signs of poor health (discharge, seizures, weight loss, lesions) were observed. It is important to note that defects may not appear until a given allele is bred to homozygosity, so researchers must be vigilant in monitoring all animals, even if no adverse effects are observed following heterozygous alterations to the genome.

### Tamoxifen dosing

One of the great innovations in the Cre-*loxP* field is the development of inducible Cre recombinase transgenic mice. However, these lines require extra experimental controls as both the inducing ligand and the recombinase may have adverse effects independent of the GOI under investigation. For example, it has been shown that induction of Cre expression alone is sufficient to generate a cardiac phenotype in the popular *α-MHC*-MerCreMer mouse line (31, 32); a major confounding effect when these mice are used to investigate the heart. Adverse effects were shown to be from the recombinase activity itself, induced by excessive tamoxifen dosing, and included apoptosis, myocardial fibrosis, and cardiovascular dysfunction (31, 32). Reassuringly, the same studies also report successful optimization of tamoxifen dosing to achieve a compromise between minimal cardiovascular side effects and maximum efficiency of recombination and gene silencing. Curiously, the ideal treatment regimen varies between strains of mice and between GOIs, making it essential for researchers who intend to work with tamoxifen to properly optimize their treatment strategy for their own lines.

### Cryopreservation of new EUCOMM mice

For most researchers, generation of genetically modified mouse lines is a significant and expensive undertaking, so line maintenance and preservation are essential. Cryopreservation of either embryos or sperm has a number of advantages over continuous maintenance of BPS at a research facility. In addition to allowing unused lines to be maintained without further breeding, which saves space and costs associated with housing and genotyping, cryopreservation provides insurance against a number of different scenarios. Loss of a mouse line can occur through infection by pathogenic organisms, which in some cases can only be eradicated by culling of the entire stock of affected animals and restocking from pre-infected sources. Genetic diversification occurs over time, through spontaneously arising mutations which can become fixed in the colony, or breeding mismanagement may also occur, leading to genetic contamination; see Brennan (16). The Jackson Laboratory, for example, routinely protects its inbred colonies against cumulative genetic drift by refreshing their breeding stocks every five generations with cryopreserved embryos. Cryopreservation also protects against the loss of a line in the most extreme circumstances, as occurred, for example, during Hurricane Katrina (37) or after an animal rights protest in Italy (38), by allowing maintenance of cryopreserved samples off-site (many cryopreservation services themselves locate samples at multiple different locations). We chose to submit our strains to the repository at the Australian Phenomics Facility, which is a founding member of the Federation of International Mouse Resources, where they have been archived by sperm cryopreservation to ensure they are widely available to the scientific community. Every line generated in this case study was submitted for cryopreservation of sperm from at least five males (mostly the first heterozygous males generated), and one straw of 11 frozen from each male was used to check for sperm concentration, morphology, and motility after thawing. For added insurance, we requested IVF confirmation of sperm viability for both *tm1a* lines, reasoning that these mice were most important because they could be reanimated to re-establish all subsequent mouse lines should individual sperm cryopreservation fail to result in recovery of a line. Of course, these are all decisions that are specific to the researchers and must fit with their goals and financial requirements.

## CRISPR/Cas9: The Way of the Future?

The CRISPR/Cas9 (clustered regularly interspaced short palindromic repeats/CRISPR-associated protein 9) system is a newly described technology that promises to be the most efficient method yet for targeting ES cells. Briefly, guiding RNA and the Cas9 endonuclease are introduced into the targeted cell (either by direct injection of the protein or as a DNA construct carrying the gene for Cas9), where the guiding RNA comprises a region of complementarity to the gene being targeted for disruption and a Cas9 binding domain. Cas9 contains two nuclease domains (a RuvC-like nuclease domain and a HNH-like nuclease domain), one for each strand of a DNA molecule, thereby introducing a double-stranded break (39). Permanent disruption of the cleaved gene results from the error-prone repair of the double stranded breaks, particularly non-homologous end-joining (NHEJ), which may result in insertions and deletions that silence the gene by introducing a frameshift. These events occur at frequencies typically above 1%, and in some cases over 50%, allowing desired mutations to be detected with simple screening assays, obviating the need for drug-resistance selection markers (40). The efficiency and simplicity of CRISPR/Cas9 may one day see this tool surpass traditional gene targeting approaches; however, there are a number of important caveats to the current application of this technology.

Off-target nuclease-mediated double-stranded breaks are the greatest concern as the “protospacer” guiding RNA region can tolerate mismatches at up to five positions – a striking number given protospacer sequences are typically 20 nucleotides in length (40). Using a relatively insensitive assay, it has been reported that nuclease-mediated indel mutations will occur in up to 5% of such mismatched off-targets (40, 41). This is an important consideration given that any 20 nucleotide sequence will have thousands of sequences differing by up to five positions in a genome.

Many approaches to improve CRISPR/Cas9 specificity have been explored. Reduced off-target effects are seen with optimization of the concentrations of both the guiding RNA and the Cas9 nuclease, by the use of multiple guiding RNAs targeted at the same gene and, most promisingly, the use of paired modified Cas9 “nickases” (39). This approach uses two guiding RNAs and two types of Cas9, one with a deactivated RuvC-like domain and the other with a deactivated HNH-like domain, meaning that each enzyme can only break one of the DNA strands on its own. Thus, the two guiding RNAs must both find their target to introduce a double-stranded break. However, it has been hypothesized that passage of a DNA replication fork through a “nick” in just one DNA strand can induce a double-stranded break, allowing the possibility of mutations arising through NHEJ (40). While screening for off-target effects is possible and necessary, it is impossible to fully assess the extent of potential genetic disruptions without completely sequencing the entire genome.

Our laboratory’s decision to create transgenic mouse lines using the EUCOMM ES cell library, instead of a CRISPR/Cas9 gene targeting system, was based simply on the availability of our GOIs as EUCOMM-targeted ES cells. Had this not been the case, we would have had more motive to explore other gene targeting solutions. Furthermore, while the Cre-*loxP* method of gene silencing is not without risk of off-target effects, we believe such effects are better characterized and more readily assessed than those associated with CRISPR/Cas9 gene targeting technology, which is to be expected given the infancy of CRISPR/Cas9 system.

### IKMC/EUCOMM or CRISPR/Cas9: Choosing the right system

CRISPR/Cas9 is clearly going to revolutionize mouse genetics, but should we abandon the IKMC/EUCOMM approach already? We would argue that both technologies need to be used in parallel, at least in the short term. If the goal is to generate knockout mice in either a global or time/tissue-specific manner, then the IKMC/EUCOMM knockout-first system should be the obvious first choice – the ES cells are most likely already archived and will enable rapid generation of each of the mice needed, as outlined above. To replicate the same mice using CRISPR/Cas9 would potentially be more difficult (although a simple global knockout is facile), as a large section of new genetic material needs to be incorporated in frame into the genome (e.g., *FRT* and *loxP* sites, *lacZ* reporter etc.). While CRISPR/Cas9 can be used to replace genetic information and to introduce new DNA, this process relies on homology-directed repair (39), which has been met with variable success. At present, CRISPR/Cas9 remains limited to smaller insertions and the availability of unique targeting sequences flanking the gene – it is not always possible to target the specific section of DNA desired using the current technology. Furthermore, the presence of the Cas9 endonuclease still subjects the genome to the risk of unintended mutations (42). New innovations in CRISPR/Cas9 technology are appearing regularly, but for now we recommend the EUCOMM knockout-first technology for generating valuable and flexible knockout mouse models.

## Conflict of Interest Statement

The authors declare that the research was conducted in the absence of any commercial or financial relationships that could be construed as a potential conflict of interest.

## Footnotes

^1^www.jaxmice.jax.org

^2^www.informatics.jax.org/recombinase.shtml

^3^www.findmice.org/summary?mutations=recombinase(cre/flp)

^4^http://www.australianphenomics.org.au/
